# Role of Glycated High Mobility Group Box-1 in Gastric Cancer

**DOI:** 10.3390/ijms22105185

**Published:** 2021-05-13

**Authors:** Shingo Kishi, Yukiko Nishiguchi, Kanya Honoki, Shiori Mori, Rina Fujiwara-Tani, Takamitsu Sasaki, Kiyomu Fujii, Isao Kawahara, Kei Goto, Chie Nakashima, Akira Kido, Yasuhito Tanaka, Yi Luo, Hiroki Kuniyasu

**Affiliations:** 1 Department of Molecular Pathology, Nara Medical University, 840 Shijo-cho, Kashihara, Nara 634-8521, Japan; nmu6429@yahoo.co.jp (S.K.); yukko10219102@yahoo.co.jp (Y.N.); m.0310.s.h5@gmail.com (S.M.); rina_fuji@naramed-u.ac.jp (R.F.-T.); takamitu@fc4.so-net.ne.jp (T.S.); toto1999-dreamtheater2006-sms@nifty.com (K.F.); isao_kawahara@a011.broada.jp (I.K.); ilgfgtk@gmail.com (K.G.); c-nakashima@naramed-u.ac.jp (C.N.); 2 Department of Orthopedics, Nara Medical University, 840 Shijo-cho, Kashihara, Nara 634-8522, Japan; kahonoki@naramed-u.ac.jp (K.H.); akirakid@naramed-u.ac.jp (A.K.); yatanaka@naramed-u.ac.jp (Y.T.); 3 Key Laboratory of Neuroregeneration of Jiangsu and Ministry of Education, Co-Innovation Center of Neuroregeneration, Nantong University, Nantong 226001, China

**Keywords:** HMGB1, glycation, *N*^ε^-(*Carboxymethyl*)*lysine*, gastric cancer

## Abstract

Advanced glycation end products (AGEs) are produced in response to a high-glucose environment and oxidative stress and exacerbate various diseases. *N*^ε^-(*Carboxymethyl*)*lysine* (CML) is an AGE that is produced by the glycation of lysine residues of proteins. There are a few reports on alterations in protein function due to CML modification; however, its association with cancer is not clear. We investigated the significance of CML modification in high mobility group box protein-1 (HMGB1), a cytokine that is significantly associated with cancer progression. Treatment of the gastric cancer cell lines TMK1 and MKN74 with glyoxal or glucose resulted in increased CML modification compared to untreated cells. CML-HMGB1 was modified via oxidation and more pronouncedly activated the receptor for AGE and downstream AKT and NF-κB compared to naïve HMGB1 and oxidized HMGB1. CML-HMGB1 bound with reduced affinity to DNA and histone H3, resulting in enhanced extranuclear translocation and extracellular secretion. Treatment of gastric cancer cells with CML-HMGB1 enhanced cell proliferation and invasion, sphere formation, and protection from thapsigargin-induced apoptosis, and decreased 5-FU sensitivity in comparison to HMGB1. Further, CML-HMGB1 was detected at various levels in all the 10 gastric cancer tumor specimens. HMGB1 levels correlated with primary tumor progression and distant metastasis, whereas CML-HMGB1 levels were associated with primary tumor progression, lymph node metastasis, distant metastasis, and stage. In addition, CML-HMGB1 levels correlated with oxidative stress in cancer tissues and resistance to neoadjuvant therapy. Therefore, CML modification of HMGB1 enhanced the cancer-promoting effect of HMGB1. In this study, CML-HMGB1 has been highlighted as a new therapeutic target, and analysis of the molecular structure of CML-HMGB1 is desired in the future.

## 1. Introduction

Gastric cancer is the third leading cause of cancer deaths in Japan [1]. Early diagnosis and early endoscopic treatment have improved the 5-year survival rate of gastric cancer patients (73.1%) [2]. However, the 5-year survival rate for advanced gastric cancer still remains poor at 47.2 and 7.3% for stages III and IV, respectively [2]. Elucidation of the mechanism of acquisition of malignant phenotypes is expected to lead to the development of novel treatments.

Taguchi et al. reported that high-mobility group box-1 (HMGB1) promotes the progression and metastasis of various cancer types [3]. We have previously reported the involvement of HMGB1 in malignancy in gastric, colorectal, prostate, and oral cancers [4,5,6,7,8,9]. Notably, the high expression of HMGB1 along with that of its receptor, i.e., receptor for advanced glycation end products (RAGE), correlates well with the progression of gastric cancer [4]. HMGB1 provides cancer cells’ proliferation, invasion, anti-apoptotic survival, and metastatic ability [10].

RAGE is a member of the immunoglobulin superfamily. It is a cell membrane receptor that has the ability to bind to multiple ligands, such as AGE, HMGB1, and S100B [11]. HMGB1 enhances oxidative stress by binding to RAGE via multiple pathways, such as mitogen-activated protein kinase, p21ras, nuclear factor (NF)-κB, AKT, phosphoinositide 3-kinase, and Wnt [12]. Importantly, RAGE also binds to AGE [13,14], which is generated at high levels in diabetes mellitus. AGE is involved in the development of complications, such as diabetic retinopathy, through RAGE activation [14,15]. We have previously reported that AGE promotes the development and progression of colorectal cancer [16,17]; it is also a risk factor for breast cancer [18].

*N*^ε^-(*Carboxymethyl*)*lysine* (*CML*) is an AGE produced by the glycation of lysine residues under physiological conditions [19]. Non-enzymatic glycation of proteins occurs through a wide range of processes, including condensation, rearrangement, fragmentation, and oxidation reactions, with Schiff bases and Amadori products as intermediates [20]. CML is one of the most abundant AGEs and correlates well with the total level of AGEs [21,22]. It has been reported that CML promotes cancer progression through RAGE in prostate, pancreatic, lung, and breast cancers [23,24,25,26,27]. HMGB1 is a 215-amino-acid-long protein that has a molecular weight of 30 kDa [28]. The HMGB1 protein possesses 44 lysine residues, which might serve as the target sites for the CML modification. We have previously reported that a high-glucose and high-fat diet increases HMGB1 levels in the colonic mucosa in a rat colon carcinogenesis model [29]. In the rats, the levels of mRNA and protein of HMGB1 were increased before forming aberrant crypt foci. In contrast, downregulation of HMGB1 inhibits cancer formation and reduces progression of the colorectal cancer [7,30,31]. Although HMGB1 has been suggested to be associated with hyperglycemic condition, glycation of HMGB1 remains poorly understood.

In this study, we examined the CML modification of HMGB1 and its significance in gastric cancer.

## 2. Results

### 2.1. CML Modification of Cancer-Related Proteins in Human Gastric Cancer Cell Lines

**Figure 1:**
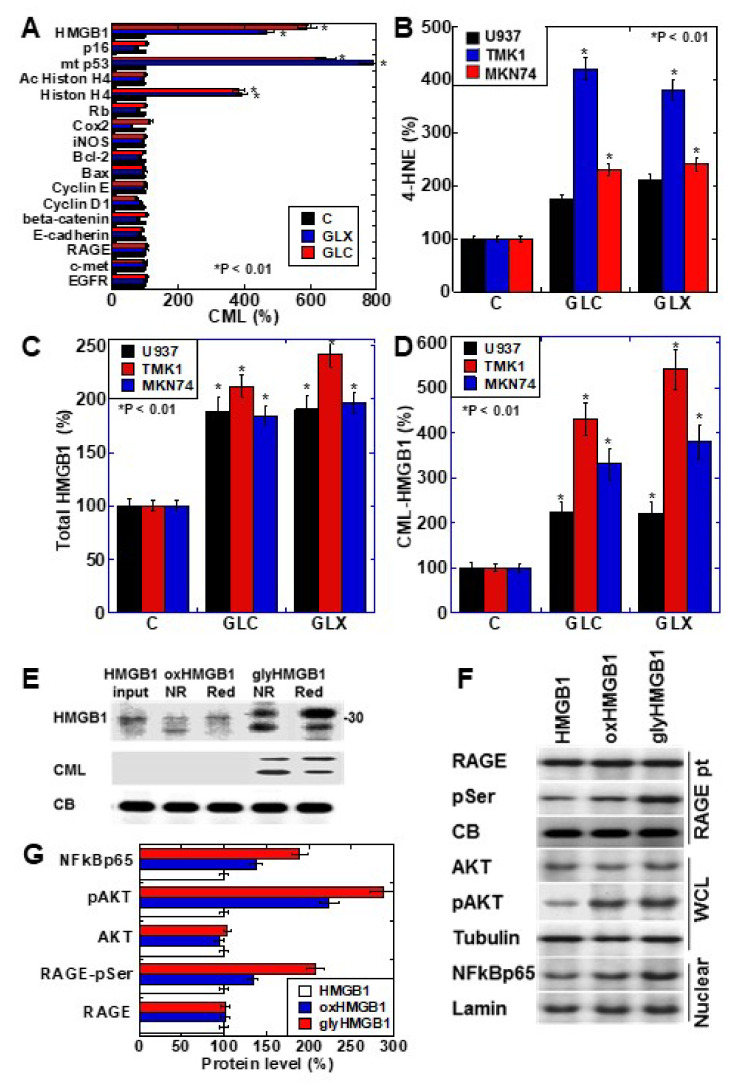
CML-HMGB1 formation in gastric cancer cells. (**A**) CML formation in various cancer-associated proteins was analyzed by antibody array using immunoprecipitant by anti-CML antibody of TMK-1 cell lysate, which was treated with GLX or GLC. The Scheme 100. (**B**) Oxidative stress levels in cells treated with GLX or GLC as measured by ELISA. (**C**) Total HMGB1 levels in cells treated with GLX or GLC as measured by ELISA. (**D**) CML-HMGB1 levels in cells treated with GLX or GLC measured by immunoprecipitation; immunoprecipitant by anti-CML antibody was detected by anti-HMGB1 antibody. (**E**) H_2_O_2_-treated HMGB1 (oxidized HMGB1) and GLX-treated HMGB1 (glycated HMGB1) were examined by Western blot analysis under non-reduced (NR) or reduced (Red) conditions. The membrane was re-probed with an anti-CML antibody (CML). (**F**) Alterations in HMGB1-associated intracellular signals in TMK-1 cells treated with naïve HMGB1 (10 μg/mL), oxidized HMGB1 (10 μg/mL), or glycated HMGB1 (10 μg/mL). (**G**) Semi-quantification of protein levels with standardization by CB, tubulin, or lamin. Phosphorylated RAGE (pSer) was examined by immunoprecipitation; RAGE precipitant was detected using an anti-pSer antibody. CB, tubulin, and lamin were used as the loading controls. Error bar and standard deviation calculated by ordinary analysis of variance from three independent experiments. CML, *N*^ε^-(*Carboxymethyl*)*lysine*; HMGB1, high-mobility group box-1; C, untreated control; GLX, glyoxal; GLC, glucose; RBP, retinol-binding protein; 4-HNE, 4-hydroxynonenal; NR, non-reduced condition; Red, reduced condition; CB, Coomassie blue; RAGE, receptor for advanced glycation end products; pSer, phosphorylated serine; pAKT, phosphorylated AKT; .NF-κB, nuclear factor-κB; RAGE pt, anti-RAGE antibody precipitant; WCL, whole cell lysate; Nuclear, nuclear fraction. * P < 0.01.

We investigated the formation of CML in cancer-related proteins after treating the human gastric cancer cell line TMK1 with glyoxal (GLX) or high-concentration glucose (GLC) (Figure 1A). The nuclear proteins HMGB1, mutant p53, and histone H4 were CML-modified by GLX and GLC treatment. Oxidative stress (read-out; 4-hyroxynonenal, 4-HNE) was increased in all the cell lines, especially TMK1 upon treatment with GLX and GLC (Figure 1B). HMGB1 concentrations in the culture medium increased in response to GLX and GLC treatment in all the cell lines, and was the highest in TMK1 (Figure 1C). GLX and GLC treatment increased the levels of CML-modified HMGB1 in the culture medium, which were higher in the culture media of TMK1 cells than in those of other cells (Figure 1D). These results suggest that CML-modified HMGB1 is extracellularly secreted. U937 is a macrophage cell line, which produces oxidative stresses as inflammatory responses. In U937 cells, GLC or GLX induced 4-HNE and total HMGB1, but CML-HMGB1 levels were lower than in gastric cancer cell lines (Figure 1B–D).

To further examine the properties of CML-HMGB1, recombinant human HMGB1 was treated with H_2_O_2_ and GLX to generate oxidized HMGB1 and glycated HMGB1, respectively (Figure 1E). Oxidized HMGB1 had higher mobility than naïve HMGB1, and under reducing conditions, the mobility of oxidized HMGB1 was similar to that of naïve HMGB1. The molecular weight of glycated HMGB1 was higher than that of naïve HMGB1; however, the mobility was lower under reducing conditions. This suggests that the SH group of HMGB1 is oxidized in glycated HMGB1. Furthermore, when the immunoblot membrane was re-probed with an anti-CML antibody after being probed with anti-HMGB1 antibody, CML was observed only in glycated HMGB1. These results indicate that HMGB1 undergoes CML modification by glycation.

Next, the effects of naïve HMGB1, oxidized HMGB1, and glycated HMGB1 on intracellular signals were examined (Figure 1F). The phosphorylation of RAGE and AKT, and the nuclear translocation of NF-κB, were more pronounced upon treatment with oxidized HMGB1 compared to those observed on treatment with naïve HMGB1; glycated HMGB1 resulted in more potent effects in this respect. Therefore, it was confirmed that glycated HMGB1 (CML-HMGB1) has a significant ability to activate intracellular signals.

### 2.2. CML Formation and HMGB1 Secretion in Human Gastric Cancer Cell Lines

**Figure 2:**
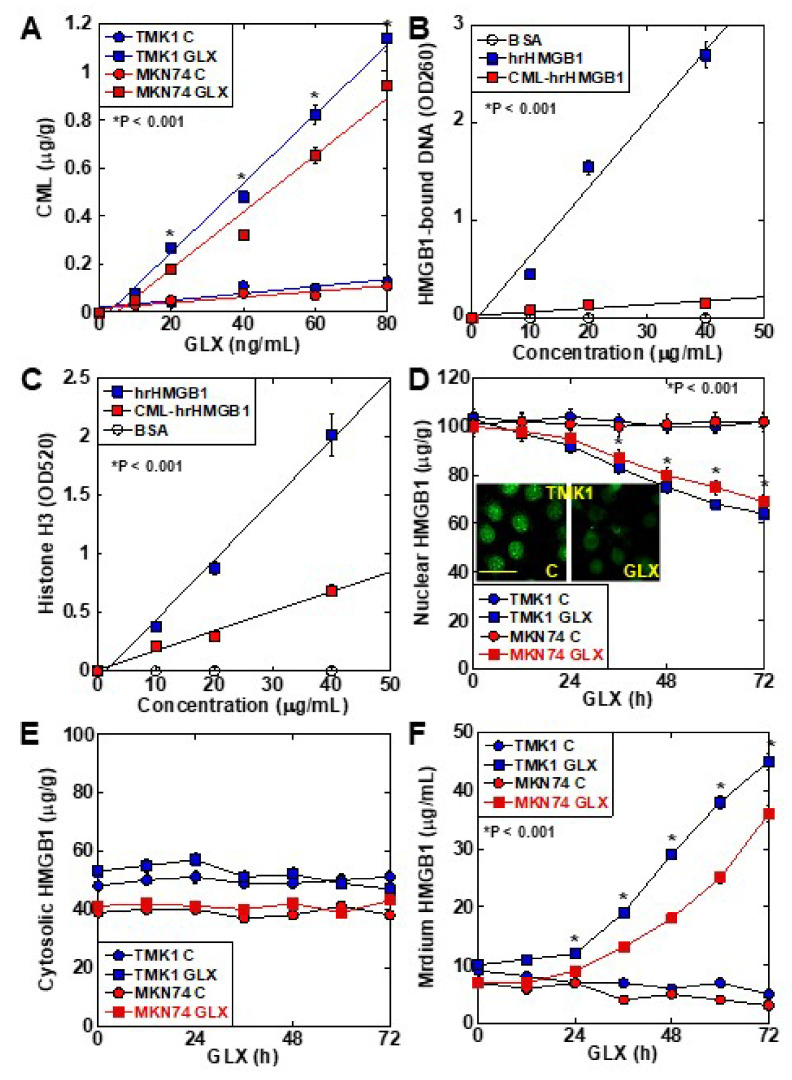
Properties of CML formation in HMGB1 in TMK-1 gastric cancer cells. (**A**) CML formation in GLX-treated TMK-1 cells measured using ELISA. (**B**) Effect of glycation on DNA binding of HMGB1 in TMK-1 cells treated with HMGB1 or GLX (CML-HMGB1). DNA extracted from the nuclear protein immunoprecipitated by HMGB1 antibody was measured using a spectrophotometer (A_260_). (**C**) Effect of glycation on histone H3 binding to rhHMGB1 or CML-rhHMGB1. rhHMGB1 or GLX-treated rhHMGB1 (CML-HMGB1) were mixed with FITC-labeled histone H3. Histone H3 was measured using a fluorescence microplate reader (A_520_). (**D**–**F**) HMGB1 in the nuclear fraction (**D**), cytosol (**E**), and cultured medium (**F**) measured by ELISA in GLX-treated TMK-1 cells. The inset of panel D shows the fluorescent immunocytochemistry of HMGB1. Scale bar, 50 μm. Error bar and standard deviation calculated by ordinary analysis of variance from three independent experiments. CML, *N*^ε^-(*Carboxymethyl*)*lysine*; HMGB1, high-mobility group box-1; hr, human recombinant; GLX, glyoxal; OD, optical density; FITC, fluorescent isothiocyanate. * P < 0.001.

CML formation occurred in a GLX concentration-dependent manner in TMK1 cells (Figure 2A). The DNA-binding ability of CML-HMGB1—produced by treating recombinant HMGB1 with GLX—was markedly decreased in comparison with that of HMGB1 (Figure 2B). Similarly, when the ability of HMGB1 to bind to histone H3 was examined (Figure 2C), a marked decrease was observed in the case of CML-HMGB1 compared to HMGB1. When TMK1 cells were treated with GLX, nuclear HMGB1 levels were reduced temporarily (Figure 2D). At this time, no significant changes were observed in HMGB1 in the cytoplasm (Figure 2E), but HMGB1 levels in the culture medium also increased (Figure 2F). These findings indicate that GLX deposited CML on nuclear HMGB1, following which the binding affinity of HMGB1 to DNA and histones in the nucleus was decreased, and HMGB1 was translocated outside the nucleus and secreted extracellularly.

### 2.3. Role of CML-HMGB1 in Gastric Cancer Cells

**Figure 3:**
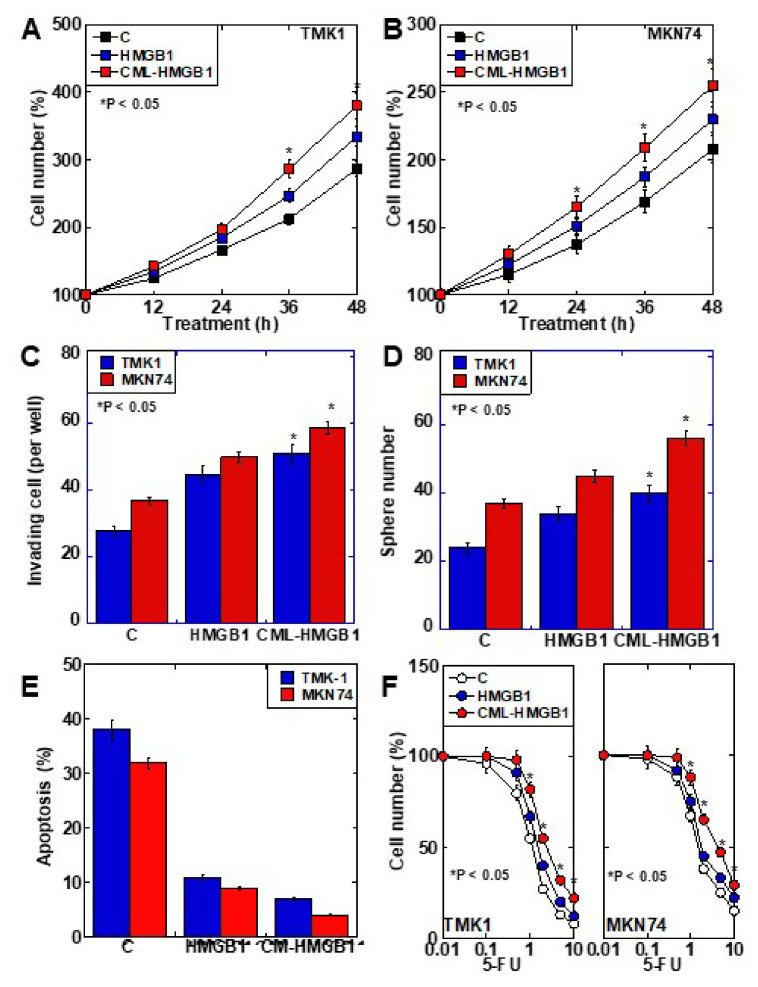
Effect of CML-HMGB1 on malignant properties of TMK-1 and MKN74 gastric cancer cells. (**A**,**B**) Effect of CML-HMGB1 (10 μg/mL) and HMGB1 (10 μg/mL) on cell proliferation. (**C**,**D**) Effect of CML-HMGB1 and HMGB1 on cell invasion by in vitro invasion assay and stemness by sphere formation. (**E**) Effect of CML-HMGB1 and HMGB1 on protection from apoptosis. Cells were untreated or treated with thapsigargin (5 μM) for 48 h with CML-HMGB1 or HMGB1. (**F**) Effect of CML-HMGB1 and HMGB1 on sensitivity to 5-FU. Cells were untreated or treated with 5-FU for 48 h with CML-HMGB1 or HMGB1 (**C**). Error bar and standard deviation calculated by ordinary analysis of variance from three independent experiments. CML, *N*^ε^-(*Carboxymethyl*)*lysine*; HMGB1, high-mobility group box-1; C, untreated control; 5-FU, 5-fluorouracil. * P < 0.05.

The role of CML-HMGB1 was compared to that of HMGB1 in the context of gastric cancer (Figure 3). First, both HMGB1 and CML-HMGB1 promoted the proliferation of TMK1 and MKN74 cell lines, but the effect of CML-HMGB1 was significantly more pronounced than that of HMGB1 (Figure 3A,B). In vitro invasion, sphere formation, and protection against thapsigargin-induced apoptosis were also promoted by HMGB1 and CML-HMGB1, but the effects were more pronounced with CML-HMGB1 (Figure 3C–E). In addition, 5-FU sensitivity (IC_50_) increased by 1.05-fold for HMGB1 and 1.3-fold for CML-HMGB1 in comparison with untreated TMK1 cells (Figure 3F). Thus, CML-HMGB1 exhibited a more significant tumor-promoting effect than HMGB1.

### 2.4. Clinical Significance of CML-HMGB1

**Figure 4:**
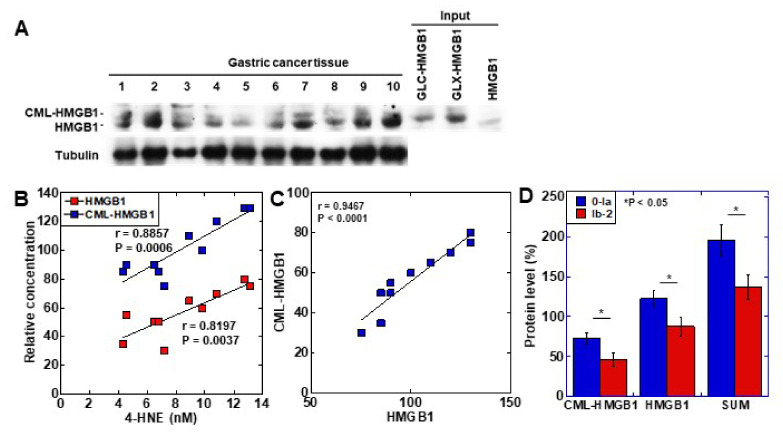
Formation of CML-HMGB1 in human gastric cancer. (**A**) CML-HMGB1 formation was examined by Western blot analysis in 10 human gastric cancer tumor tissues. (**B**) Comparison of HMGB1 and CML-HMGB1 levels with oxidative stress (4-HNE levels). The correlation was calculated using the Pearson’s correlation test. (**C**) Association between the relative levels of HMGB1 and CML-HMGB1. The correlation was calculated using the Pearson’s correlation test. (**D**) Effect of CML-HMGB1 on drug resistance in gastric cancer patients. Histological grade of therapeutic effect was determined according to Japanese Gastric Cancer Classification [32]. Grade 0, no effect; Grade 1a, very slight effect; Grade 1b, slight effect; Grade 2, considerable effect. Error bar, standard deviation calculated by ordinary analysis of variance from three independent experiments. CML, *N*^ε^-(*Carboxymethyl*)*lysine*; HMGB1, high-mobility group box-1;.4-HNE, 4-hydroxynonenal; SUM, summation of HMGB1 and CML-HMGB1 protein levels. * P < 0.05.

To examine the clinical significance of CML-HMGB1, the protein level of CML-HMGB1 was examined by Western blotting in 10 gastric cancer samples (Figure 4A). CML-HMGB1 was detected at various levels in all the samples. The protein levels of CML-HMGB1 and HMGB1 were semi-quantified and a correlation with clinicopathological parameters was investigated (Table 1). HMGB1 levels were correlated with T factor (primary tumor) and M factor (distant metastasis), whereas CML-HMGB1 levels were correlated with all T, N (nodal metastasis), and M factors, and stage. No correlation was found with grade for either CML-HMGB1 or HMGB1.

The levels of the oxidative stress marker 4-HNE were measured in the same tissue specimens and compared to those of CML-HMGB1 and HMGB1 (Figure 4B). Both CML-HMGB1 and HMGB1 levels correlated with 4-HNE, but CML-HMGB1 exhibited a more significant correlation with oxidative stress (*p* = 0.0006 vs. *p* = 0.0037). A correlation was identified between the levels of CML-HMGB1 and HMGB1 (*p* < 0.0001) (Figure 4C).

As HMGB1 was found to be correlated with drug resistance [33], we examined the relationship between drug sensitivity and protein levels of CML-HMGB1 or HMGB1 in the above 10 samples (Figure 4D). CML-HMGB1 and HMGB1—and the sum of both—were found to be expressed at higher levels in the insensitive group (grade 0–1a) than in the sensitive group (grade 1b–2). By multivariate analysis (Table 2), CML-HMGB1 showed a less significant correlation with drug resistance than HMGB1; however, summation of HMGB1 and CML-HMGB1 showed more significant correlation with drug resistance than HMGB1 alone. This suggests that CML-HMGB1 promotes drug resistance accompanying HMGB1.

## 3. Discussion

In the present study, CML was deposited on HMGB1 protein molecules associated with oxidative stress. CML-HMGB1 activated cancer-associated signals, such as RAGE, AKT, and NF-κB, exceeding the effect of oxidized HMGB1, and promoted proliferation, invasion, survival, stemness, and drug resistance development. CML-HMGB1 was also produced in gastric cancer tissues and was found to be associated with cancer progression and drug resistance.

We examined the intracellular proteins that were susceptible to glycation (Figure 1A). Among the proteins examined, proteins with a relatively long half-life were found to be glycated. Histones have a half-life of more than 150 days [34,35]. It is known that glycation occurs in proteins with a long half-life such as collagen [36] and superoxide dismutase (SOD) [37], which are strongly exposed to oxidative stress. The half-life of the wild-type p53 protein is as short as 10 min, but it is prolonged in the mutant [38]. TMK1 cells express mutated p53 [39], and exhibit high levels of CML modification. HMGB1 has a half-life of 18 h [40]; however, HMGB1 has 44 lysine residues (of a total of 215 amino acid residues). In contrast, histone H4 contains 11 lysine residues (of a total of 103 amino acid residues). Therefore, it is thought that the presence of so many lysine residues in HMGB1 makes it susceptible to glycation.

To date, it has been reported that glycation reduces protein function. Collagen glycation results in cross-linking, which strongly impairs fiber–fiber or fibril–fibril sliding and reduces tissue viscoelasticity [36]. Glycation induces two-dimensional or three-dimensional structural changes in SOD, resulting in the loss of enzyme activity [37]. As a non-histone chromosomal protein, HMGB1 stabilizes the nucleosomes and regulates the expression of several genes [41]. It is also involved in DNA repair and replication [42]. Our data suggest that glycated HMGB1 binds to DNA and histones with reduced affinity, thereby altering functions such as DNA repair, replication, and transcription. The DNA-binding ability of HMGB1 and its effect on DNA bending are regulated by the degree of acetylation of lysine residues in HMGB1 protein. CML modification on HMGB1 lysine residues is thought to inhibit lysine acetylation [43]. In addition, our experiments suggest that the reduced integration of HMGB1 within the nucleosome resulted in its translocation from the nucleus to the cytoplasm and extracellular space. Acetylation at two HMGB1 nuclear localization signals results in inhibited nuclear translocation of HMGB1 and induces cytoplasmic accumulation [41]. It is also possible that CML deposition at HMGB1 nuclear localization signals might impair the nuclear translocation of HMGB1. The promotion of HMGB1 secretion by glycation suggests that a glycation-promoting condition, such as diabetes, might increase the extracellular HMGB1 levels. We have reported that a high-sugar and high-fat diet promotes HMGB1 secretion in rat colonic mucosa in a rodent colon carcinogenesis model [16,29].

In all, we believe that glycation impairs the function of HMGB1 as a nuclear protein. CML-HMGB1 induced more pronounced phosphorylation of serine residues in RAGE [44], and greater activation of intracellular signals AKT and NFκB, than HMGB1. It is expected that the CML modification of the KKKK sequence between the C-terminal DNA-binding domain and the RAGE-binding domain on the N-terminal side might alter the three-dimensional structure of HMGB1, resulting in altered affinity for RAGE.

Both HMGB1 and AGE bind to RAGE [12]. We have previously reported the differences between HMGB1 and AGE as RAGE ligands [5]. A difference was found in VEGF induction between the two groups. However, no studies on glycated HMGB1 have been reported. We previously described that the effects of HMGB1 are enhanced in an AGE-forming environment. In an azoxymethane rat colon carcinogenesis model fed glucose and linoleic acid, AGE formation promoted the expression of RAGE and HMGB1 and enhanced carcinogenesis [16,29]. In diabetic conditions, HMGB1 may be glycated. Furthermore, in colorectal cancer with diabetes, hemoglobin A1c levels are correlated with cancer stage and metastasis [17], and it has been reported that liver metastasis is particularly promoted in diabetes-complicated colorectal cancer [45]. Data from our small cohort of gastric cancer patients show that HMGB1 levels were correlated with primary tumor progression and distant metastasis, whereas CML-HMGB1 levels were correlated with primary tumor progression, lymph node metastasis, distant metastasis, and stage. Therefore, it is suggested that CML-HMGB1 might be more significantly involved in gastric cancer progression than HMGB1.

In recent years, it has been reported that the oxidation of intramolecular cystine residues is important for HMGB1 activation [46]. Oxidation of C23 and C45, but not C106, results in the formation of an S–S bond between these residues (partially oxidized HMGB1), which provides a feature that exhibits a strong pro-inflammatory effect [47]. In contrast, our data show that glycated HMGB1 can more strongly promote cell proliferation, infiltration, survival, stemness, and drug resistance development than oxidized HMGB1. Interestingly, it has been suggested that glycated HMGB1 undergoes oxidation. Glycation is promoted by oxidative stress [27]. In our study, glycated HMGB1 levels correlated with 4-HNE levels in both gastric cancer specimens and in vitro glycated cell lines. HMGB1 protein levels were also correlated with 4-HNE levels; however, glycated HMGB1 levels were more strongly correlated with 4-HNE levels. These findings suggest that oxidative stress promotes the secretion of HMGB1 as well as the production of glycated HMGB1 and is involved in increasing the malignancy of gastric cancer.

Our study revealed that CML-HMGB1 correlates with oxidized HMGB1 and promotes cancer malignancy more than oxidized HMGB1, but its structural differences and receptor affinity have not been clarified. It is also necessary to investigate which of the lysine residues of HMGB1 are affected by CML modification, leading to the alteration in its function.

In this study, we reported that glycation of HMGB1 enhances the cancer-promoting effect of HMGB1. Glycated HMGB1 attracts attention as a molecule with new bioactivity and might be a marker for cancer malignancy or a new therapeutic target. Furthermore, it is suggested that examining the effects of glycation on more protein molecules might lead to the elucidation of disease mechanisms in cancer, diabetes, degenerative diseases, and aging.

## 4. Materials and Methods

### 4.1. Cell Lines and Reagents

The human gastric carcinoma cell lines TMK1 and MKN74 were gifted by Professor Wataru Yasui (Molecular Pathology, Hiroshima University, Hiroshima, Japan). The human monocytic cell line U937 was purchased from Dainihon Pharmacy Co. (Tokyo, Japan). TMK1 and MKN74 cells were cultured in Dulbecco’s modified Eagle medium (DMEM; Wako Pure Chemical Corporation, Osaka, Japan) supplemented with 10% fetal bovine serum (FBS; Sigma, St. Louis, MO, USA) at 37 °C in 5% CO_2_. U937 cells were cultured in RPMI-1640 (Wako) supplemented with 10% FBS (Sigma) at 37 °C in 5% CO_2_. For in vitro glycation, cells were treated with D-glucose (50 mM, Sigma) or glyoxal (50 μM, Sigma) in DMEM for 7 d and 24 h, respectively.

### 4.2. Patients

We obtained frozen tissue samples from 10 patients with gastric cancer, involving those who were treated by surgical resection with neoadjuvant chemotherapy at the Nara Medical University Hospital. The tissues were subjected to a histopathological review at the Department of Molecular Pathology, Nara Medical University, during 2001–2019. As written informed consent was not obtained from the patients for their participation in the present study, all identifying information was removed from patient samples prior to their analysis to ensure strict privacy protection (unlinkable anonymization). All procedures were performed in accordance with the Ethical Guidelines for Human Genome/Gene Research enacted by the Japanese Government and with the approval of the Ethics Committee of Nara Medical University (approval number: 937).

### 4.3. Glycation and Oxidization of HMGB1

For glycation of HMGB1, recombinant human HMGB1 (50 μg, R&D Systems, Minneapolis, MN, USA) was incubated with D-glucose (GLC; Sigma, 500 mg/dL, for 48 h) or glyoxal (GLX; Sigma, 1 μM, for 6 h) at room temperature. For oxidization of HMGB1, recombinant human HMGB1 (50 μg, R&D Systems) was incubated with 100 μL of 50 μM H_2_O_2_ (Wako) on ice for 1 h. Glycated HMGB1 and oxidized HMGB1 were dialyzed using a dialysis membrane (Thermo Fisher Scientific, Tokyo, Japan) with phosphate-buffered saline (PBS, 2L) at 4 °C for 6 h, twice, to remove unreacted glucose or glyoxal.

### 4.4. Cell Growth and Apoptosis

Cell growth was assessed using the 3-(4,5-dimethylthiazol-2-yl)-5-(3-carboxymethoxyphenyl)-2-(4-sulfophenyl)-2H-tetrazolium (MTS)-based Celltiter 96 Aqueous One Solution Cell Proliferation Assay kit (Promega Biosciences Inc., San Louis Obispo, CA, USA), as previously described [48]. The absorbance was measured at 490 nm on a Multiskan FC Microplate Photometer (Thermo Fisher). Apoptosis was induced by 5 μM thapsigargin, as previously described [49]. Briefly, a total of 1000 cells were stained with Hoechst 33342 dye (Life Technologies, Carlsbad, CA, USA), and examined under a fluorescent microscope [4].

### 4.5. Chamber Invasion Assay

A modified Boyden chamber assay was used to examine the in vitro invasion of cancer cells [50]. Following incubation at 37 °C for 24 h, the filters were carefully removed from the inserts, stained with hematoxylin for 10 min, and mounted on microscopic slides. The number of stained cells in each insert was counted at 100× magnification. Invasion activity was quantified by calculating the average number of cells per well. All experiments were performed in triplicate.

### 4.6. Sphere Formation Assay

Cells (1000 cells per well) were seeded in uncoated bacteriological 35-mm dishes (Coning Inc., Corning, NY, USA) with 3D Tumorsphere Medium XF (Sigma) [48]. Following culturing for 7 d, sphere images were taken by an inverted microscope coupled with a camera. The images were captured on a computer, and the sphere number was measured using NIH ImageJ software (version 1.52, Bethesda, MD, USA).

### 4.7. Protein Extraction

To prepare whole cell lysates, cells were washed twice with cold PBS, harvested, and lysed with 0.1% SDS-added RIPA buffer (Thermo Fisher) [4]. Cell fractions were extracted by processing the cells with a Cell Fractionation Kit (Abcam, Cambridge, UK), according to the manufacturer’s instructions [51]. Protein assays were performed using a Protein Assay Rapid Kit (Wako).

### 4.8. Immunoprecipitation

For immunoprecipitation, the lysates were incubated with antibodies against CML (Abcam) or RAGE (Santa Cruz Biotechnology, Inc., Santa Cruz, CA, USA) and protein A/G agarose (Santa Cruz) for 3 h at 4 °C. Precipitates were collected by centrifugation, washed five times with lysis buffer, and investigated using immunoblot analysis and protein antibody array.

### 4.9. Immunoblot Analysis

Whole cell lysates were prepared as previously described [4]. Lysates (50 μg) were subjected to immunoblot analysis in 12.5% sodium dodecyl sulfate-polyacrylamide gels, followed by electrotransfer onto nitrocellulose membranes (Bio-Rad, Hercules, CA, USA). The filters were incubated with primary antibodies and then with peroxidase-conjugated IgG antibodies (MBL, Nagoya, Japan). Antibodies against tubulin (Oncogene Research Products, Cambridge, MA, USA) or lamin (Proteintech Group Inc., Rosemont, IL, USA) were used to assess the levels of protein loaded per lane. The immune complex was visualized using a CSA system (DAKO, Carpinteria, CA, USA). Antibodies against HMGB1 (Proteintech), RAGE, AKT, phosphorylated AKT (Santa Cruz), phosphorylated serine (pSer), CML (Abcam), and NF-κB p65 (Biorbyt LLC, St Louis, MO, USA) were used as the primary antibodies.

### 4.10. Protein Antibody Array

Antibody (2 μg in 200 μL PBS) was dot-blotted onto cellulose membranes using a 96-well dot-blot apparatus (Bio-Rad). The antibodies used for the assay are listed in Table 3. The membranes were incubated with total immunoprecipitates of anti-CML antibody (10 μg) or cell lysate (50 μg) in 5 mL of Tris-buffered saline (TBS, Wako) with 0.1% bovine serum albumin (Sigma) and 0.05% Tween 20 (Wako) at room temperature for 6 h. The membrane was then washed three times with TBS containing 0.05% Tween 20. The membranes were stained with a Silver Stain 2 Kit (Wako). Signal intensities were measured using the NIH ImageJ software (version 1.52). The signal ratio of the immunoprecipitates to the lysate for each antibody is shown in Figure 1A. For standardization, the signal ratio of immunoprecipitates to lysate on retinol-binding protein (RBP) in untreated cells was set to 100, which is a rapid turnover protein that hardly yields glycation [52].

### 4.11. Reverse Transcription-Polymerase Chain Reaction (RT-PCR)

To assess human and murine mRNA expression, RT-PCR was performed with 0.5 µg total RNA extracted from the three cell lines using the RNeasy kit (Qiagen, Germantown, MD, USA). The primer sets are listed in Table 1 and were synthesized by Sigma Genosys (Ishikari, Japan). PCR products were electrophoresed on a 2% agarose gel and stained with ethidium bromide. *GAPDH* mRNA was also amplified for use as an internal control.

### 4.12. Enzyme-Linked Immunosorbent Assay (ELISA) and Fluorometric Assay

An ELISA kit (Abcam, Cambridge, MA, USA) was used to measure the concentration of 4-hydroxynonenal (4-HNE). The assay was performed according to the manufacturer’s instructions, and whole cell lysates were used for the measurements.

### 4.13. HMGB1-Bound DNA

Cells were suspended in hypotonic buffer (0.1 mM piperazine-N,N’-bis(2-ethane sulfonic acid) (PIPES) pH 6.5, 5 mM CaCl_2_, 5 mM dithiothreitol (DTT), 0.5% Triton X-100, 0.5% NP-40), and centrifuged at 1000× *g*. Cell pellets were sonicated in extraction buffer (0.35 M NaCl, 20 mM Tris-HCl pH 7.2, 12 mM MgCl_2_, 5 mM ethylene glycol bis(2-aminoethyl ether)tetraacetic acid (EGTA), 5 mM DTT) for 1 min three times on ice. The nuclear extract was incubated with HMGB1 antibody (Proteintech) and protein A/G agarose (Santa Cruz) for 3 h at 4 °C. Precipitates were collected by centrifugation and washed five times with lysis buffer. The DNA concentration (A_260_) of the precipitate solution was measured using a spectrophotometer (Beckman Coulter Life Sciences, Indianapolis, IN, USA).

### 4.14. Histone Binding Ability of HMGB1

Fluorescein isothiocyanate (FITC)-labeled human recombinant histone H3 (2 μg, Rockland Immunochemicals Inc., Limerick, PA, USA) was mixed with glycated HMGB1 (2 μg) or human recombinant HMGB1 in Tris-buffered saline (100 μL) and incubated at 4 °C for 12 h. The mixture solution was immunoprecipitated using an anti-HMGB1 antibody (Proteintech) and protein A/G agarose (Santa Cruz) for 3 h at 4 °C. Precipitates were collected by centrifugation and washed five times with lysis buffer. The fluorescence intensity (A_520_) of histone H3 in the precipitate solution was measured using a fluorescence microplate reader (Thermo Fisher).

### 4.15. Statistical Analysis

Statistical significance was calculated using a two-tailed Fisher’s exact test, an ordinary analysis of variance, and InStat software (GraphPad, Los Angeles, CA, USA). Correlations were tested using Pearson’s correlation test. A two-sided *p* value of < 0.05 was considered to indicate statistical significance. Multivariate analysis was done by multiple comparison analysis.

## 5. Conclusions

**Figure 5:**
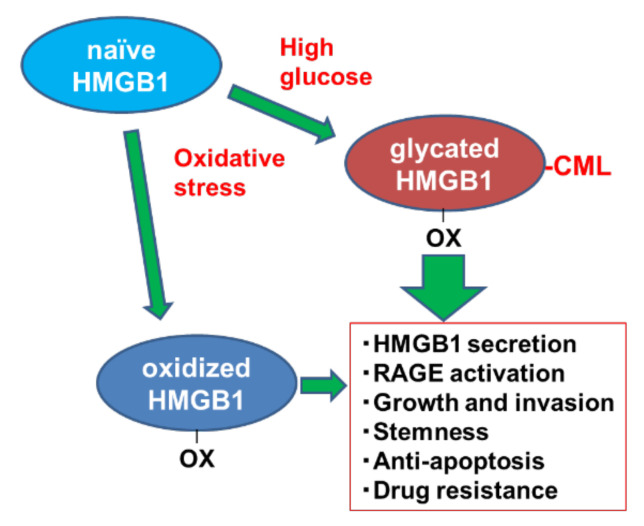
Conclusion of this study. Glycated HMGB1 modified from naïve HMGB1 by high-glucose conditions and oxidative stress enhances pro-tumoral features. HMGB1, high mobility group box-1; OX, oxidized cysteine residue; CML, *N*^ε^*-(Carboxymethyl*)*lysine*; RAGE, receptor for glycated end products.

As shown in Figure 5, naïve HMGB1 is modified to glycated HMGB1 by high-glucose conditions and oxidative stress. Glycated HMGB1 enhances extranuclear translocation and secretion of HMGB1, activation of intracellular signaling of RAGE, AKT and NFκB, cell growth, invasion ability, stemness, anti-apoptotic survival, and drug resistance. Such tumor-promoting properties are common to HMGB1 (oxidative HMGB1); however, glycated HMGB1 is more pronounced, and it is thought that both types of HMGB1 might promote cancer in a cooperating manner.

## Figures and Tables

**Table 1:** Relationship between pathological parameters and protein levels of HMGB1 or CML-HMGB1 in gastric cancer samples.

Parameter ^1^	Level	Number	Protein Level			
			HMGB1 ^2^	*p* ^3^	CML-HMGB1 ^2^	*p* ^3^
Grade	1	6	104 ± 21	NS	59 ± 17	NS
	2	4	98 ± 21		54 ± 18	
pT	2	3	8 ± 3	0.0015	43 ± 12	0.0245
	3	4	96 ± 11		54 ± 13	
	4	3	127 ± 8		75 ± 2	
pN	0	3	83 ± 8	NS	38 ± 10	0.0069
	1 and 2	7	109 ± 10		65 ± 10	
pM	0	8	94 ± 4	0.0123	52 ± 14	0.0377
	1	2	130 ± 7		78 ± 4	
pStage	1 and 2	3	83 ± 8	NS	38 ± 10	0.0069
	3 and 4	7	109 ± 18		65 ± 11	

^1^ Histological and clinicopathological classification were according to Japanese Gastric Cancer Classification [32]. pT1, tumor confined to the mucosa or the submucosa; pT2, tumor invades the muscularis propria; pT3, tumor invades the subserosa; pT4, tumor invasion is contiguous to or exposed beyond the serosa or tumor invades adjacent structures; pN0, no regional lymph node metastasis; pN1, metastasis in 1–2 regional lymph nodes; pN2-3, metastasis in 3 or more regional lymph nodes; M0, no distant metastasis; M1, distant metastasis; pStage I, pT1/pN0, pT1/pN1, or pT2/pN0; pStage II, pT1/pN2–3, pT2/pN1–2, or pT3/pN0; pStage III, pT2/pN3, pT3pN2–3, or pT4/pT1–3; pStage IV, any T/any N/M1. ^2^ Protein levels were calculated from the signal intensities of Western blot analysis (Figure 4A). ^3^ Statistical significance was tested using an ordinary analysis of variance. NS, not significant.

**Table 2:** Multivariate analysis of HMGB1, CML-HMGB1, and the sum for drug resistance.

Variable	Coefficient	SE	95% Confidence Interval	*p*
HMGB1	−0.01398	0.01444	−0.04812 to 0.02016	0.0014
CML-HMGB1	0.03455	0.01189	0.006428 to 0.06267	
SUM	−1.612	0.3951	−2.523 to −0.7007	0.0008

Multiple regression analysis was performed for multivariate analysis.

**Table 3:** List of antibodies used for the antibody array.

Protein	Company	Cat #
HMGB1	Abcam	ab18256
p16	Santa Cruz	SC-377412
mut p53	Abcam	ab32509
Ac Histone H4	Rockland	600-401-P06
Histone H4	Santa Cruz	SC-25260
Rb	Santa Cruz	SC-74562
Cox2	Santa Cruz	SC-514489
iNOS	Santa Cruz	SC-7271
BCL-2	Proteintech	26593-1-AP
BAX	Proteintech	50599-2-IG
Cyclin E	Proteintech	11554-1-AP
Cyclin D1	Proteintech	26939-1-AP
β-catenin	Proteintech	13665-1-AP
E-cadherin	Santa Cruz	SC-8426
RAGE	Santa Cruz	SC-365154
c-met	Life Span	LS-C826326-100
EGFR	Santa Cruz	SC-373746
RBP	Abcam	ab48624

HMGB1, high-mobility group box-1; mut, mutated; Ac, acetylated; Rb, retinoblastoma; Cox2, cyclooxygenase-2; iNOS, inducible nitric oxide synthase; BCL-2, B-cell lymphoma-2; BAX, Bcl-2-associated X protein; RAGE, receptor for advanced glycation end products; EGFR, epithelial growth factor receptor; RBP, retinol-binding protein.

## Data Availability

Not applicable.

## Abbreviations

AGE	Advanced glycation end products
CML	*N*^ε^-(*Carboxymethyl*)*lysine*
HMGB1	high mobility group box protein-1
RAGE	receptor for advanced glycation end products
NF	nuclear factor
GLX	glyoxal
GLC	glucose
SOD	superoxide dismutase
